# Vascular Endothelial Growth Factor (VEGF) Detection Using an Aptamer and PNA-Based Bound/Free Separation System

**DOI:** 10.3390/ma7021046

**Published:** 2014-02-11

**Authors:** Chifuku Mita, Koichi Abe, Takahiro Fukaya, Kazunori Ikebukuro

**Affiliations:** Department of Biotechnology and Life Science, Tokyo University of Agriculture and Technology, 2-24-16 Naka-cho, Koganei, Tokyo 184-8588, Japan; E-Mails: 50012641130@st.tuat.ac.jp (C.M.); abekou@cc.tuat.ac.jp (K.A.); t.fukaya87@gmail.com (T.F.)

**Keywords:** aptamer, bound free separation, peptide nucleic acid

## Abstract

We have developed a bound/free separation system using a vascular endothelial growth factor (VEGF) aptamer and a peptide nucleic acid (PNA) to detect VEGF. In this system, we designed capture PNA (CaPNA), which hybridizes with the aptamer in the absence of the target protein, but does not hybridize with the aptamer in the presence of the target protein due to steric hindrance and/or stabilization of the aptamer’s structure. By removing the aptamers not bound to the target protein using CaPNA immobilized beads, we can detect the target protein by measuring signals labeled with the aptamer in the supernatant. In this study, we detected VEGF using CaPNA-immobilized beads without the time-consuming washing step. This simple and rapid system can detect 25 nM of VEGF in 15 min.

## 1. Introduction

Point-of-care testing (POCT), which can produce diagnostic results at the bedside of patients, allows for the selection of the most appropriate treatment, leading to an improved clinical or economic outcome. Therefore, POCT systems should be compact, simple, inexpensive, and provide rapid results.

Aptamers are attractive molecular recognition elements for POCT because they can be synthesized and modified with any molecule at a lower cost than antibodies. Various aptamer-based sensing systems have been reported (reviewed in [1,2]). Using the complementary DNA of an aptamer, we can construct unique, versatile sensing systems. We have previously described an aptamer-based bound/free separation (B/F separation) system using complementary capture DNA (CaDNA) [3,4]. Target binding to the aptamer stabilizes the aptamer structure, which then inhibits the hybridization of complementary DNA to the aptamer. Immobilized complementary DNA can remove excess fluorescent or enzyme-labeled aptamers from the solution upon target binding, allowing B/F separation and detection of aptamer-target molecule complexes by measuring the signal of the labeled aptamer. However, complementary DNA hybridization is slow due to negative charge repulsion. In this study, we used a peptide nucleic acid (PNA) to construct a rapid biomarker detection system using an aptamer.

PNA is a synthetic DNA mimic that can hybridize to complementary DNA or RNA strands. In PNA, the negatively charged phosphate backbone of nucleic acids is replaced by an uncharged *N*-(2-aminoethyl)-glycine scaffold. The uncharged nature of PNA is responsible for the better thermal stability of PNA-DNA duplexes compared with that of DNA-DNA duplexes, because of the lack of electrostatic repulsion between the two strands [5,6]. Based on these advantages, some aptameric sensors combined with PNA have been reported [7–9]. Here, our goal was to construct a rapid aptamer-based B/F separation system using PNA, because PNA can hybridize with DNA more rapidly than complementary DNA [10].

**Figure 1.**
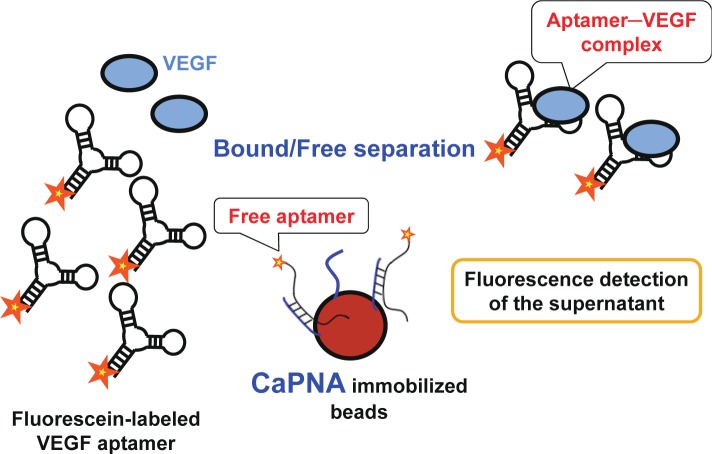
Scheme of the single aptamer-based bound/free separation system using captured peptide nucleic acids (CaPNAs). The fluorescein-labeled aptamer was incubated with target proteins. The mixture was then incubated with CaPNA-immobilized beads. After centrifugation, the fluorescence intensity of the supernatant was measured. In the absence of target molecules, the fluorescein-labeled aptamers were captured by CaPNA immobilized beads, resulting in a decrease in the fluorescence intensity. When the target molecule binds to the aptamer, hybridization between the aptamer and the CaPNA is inhibited due to stabilization of the aptamer structure and/or steric hindrance via binding of the target molecule, resulting in an increase in the fluorescence intensity of the supernatant.

In this study, we developed a detection system sensing vascular endothelial growth factor (VEGF)—which is one of the most important biomarker candidates for cancers [11,12]—as a model, using a VEGF aptamer that we previously obtained (publication in progress) and capture PNA (CaPNA) that is complementary to part of the aptamer. Since the thermal stability of PNA-DNA duplexes is higher than that of DNA-DNA duplexes, we expected VEGF to be detected more rapidly when using PNA instead of DNA. The sensing scheme using CaPNA is shown in Figure 1. In the absence of VEGF, CaPNA can hybridize with the aptamer, whereas it does not readily do so with the aptamer–VEGF complex in the presence of VEGF, because the structure of the VEGF aptamer is stabilized upon VEGF binding, and because VEGF inhibits CaPNA hybridization due to steric hindrance. Therefore, the aptamer is not trapped by CaPNA-immobilized beads when bound to VEGF. To detect aptamer-VEGF complexes in the supernatant, the aptamer was labeled with fluorescein. As such, VEGF can be rapidly detected with the B/F separation step using a single aptamer.

## 2. Results and Discussion

**Figure 2.**
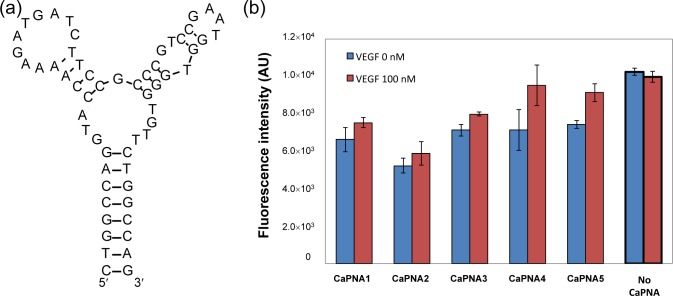
(**a**) Predicted secondary structure of the VEGF aptamer used in this study; (**b**) Determination of effective CaPNAs. The fluorescein-labeled VEGF aptamer was incubated with VEGF (100 nM). Each CaPNA-immobilized bead was incubated with the VEGF-aptamer mixture. The fluorescence intensity of the supernatant after centrifugation was measured. The blue bar shows the fluorescence intensity in the absence of VEGF. The red bar shows the fluorescence intensity in the presence of VEGF. No CaPNA indicates the fluorescence intensity using a bead that CaPNA has not immobilized.

We designed CaPNAs that hybridize with the VEGF aptamer only in the absence of VEGF (Table 1). We used a 58-mer VEGF aptamer (Figure 2a) with a three-way junction predicted secondary structure. The *T*_m_ value of the secondary structure of the VEGF aptamer is 47°C, calculated by the mfold web server [13]. Previous mutational analysis has indicated that the predicted second stem-loop structure of the aptamer is important for VEGF binding. Therefore, we hypothesized that binding of VEGF to the aptamer would efficiently inhibit hybridization of the complementary PNA with the second stem-loop region of the aptamer. We designed five CaPNA candidates that are complementary to the sequence surrounding the predicted second stem–loop sequence of the aptamer with higher *T*_m_ values than the predicted *T*_m_ value of the VEGF aptamer. The five CaPNAs (CaPNA1, 2, 3, 4, and 5) have different lengths (11, 11, 10, 9, and 8-mer, respectively). CaPNA1 is complementary to the central region of the second stem-loop. CaPNA2 is complementary to the 3´ terminal region of the second stem-loop. CaPNA3, 4, and 5 are complementary to the 5´ terminal region of the second stem-loop sequence. CaPNA4 and 5 have a deletion of one and two base(s), respectively, compared to CaPNA3. The *T*_m_ values for hybridization of CaPNA1–5 to the aptamer were 62, 59, 65, 57, and 47°C, respectively, as calculated by the PNA probe designer [14].

We evaluated the capture efficiency of the designed CaPNAs (Figure 2b). The lowest fluorescence intensity was observed when CaPNA2 was incubated with the supernatant in the absence of VEGF, indicating that, among the five designed CaPNAs, CaPNA2 hybridizes with the VEGF aptamer most effectively. However, the fluorescence intensity also decreased in the presence of VEGF when compared with that of the supernatant not incubated with CaPNA2 immobilized beads, which indicates CaPNA2 binds to the VEGF aptamer in the presence and absence of VEGF. CaPNA2 might bind to the VEGF-aptamer complex or bind to the VEGF aptamer more strongly than VEGF. CaPNA1 and CaPNA3 showed similar fluorescence intensity. CaPNA4 and CaPNA5 presented similar fluorescence intensity as CaPNA1 and CaPNA3 in the absence of VEGF, but CaPNA4 and CaPNA5 showed an increase in the fluorescence intensity in the presence of VEGF compared to in its absence. We selected CaPNA4 for subsequent experiments because the difference between its fluorescence intensities in the absence and presence of VEGF was the largest among the five CaPNAs.

**Figure 3.**
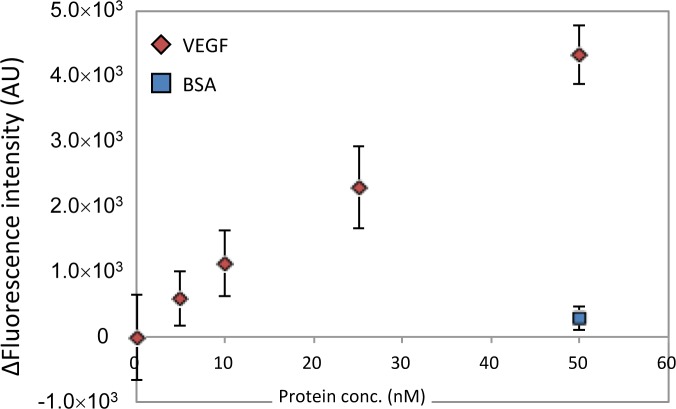
Calibration graph for VEGF using aptamer-based B/F separation with CaPNA4. The fluorescein-labeled VEGF aptamer (10 nM) and CaPNA4-immobilized beads (10 μL) were used. The fluorescence intensity of the supernatant was measured (mean ± SD; *n* = 3). The red diamonds show the fluorescence intensity in the presence of each concentration of VEGF. The blue diamond shows the fluorescence intensity in the presence of BSA (50 nM).

Next, we evaluated the concentration dependency of VEGF detection using CaPNA4-immobilized beads over a short time period. The VEGF aptamer and VEGF were incubated for 10 min, and the CaPNA4-immobilized beads and VEGF solution were incubated for 5 min. We observed an increase in the fluorescence intensity in a VEGF concentration-dependent manner (Figure 3). The magnitude of the fluorescence intensity depended on the VEGF concentration within the range of 5 nM to 50 nM, and the detection limit was 25 nM VEGF (*S*/*N* = 3). However, a slight increase in the fluorescence intensity was observed in the presence of bovine serum albumin (BSA). These data indicate that a concentration of 25 nM VEGF can be detected using the fluorescein-labeled VEGF aptamer and CaPNA4-immobilized beads in 15 min with a single B/F separation step.

**Figure 4.**
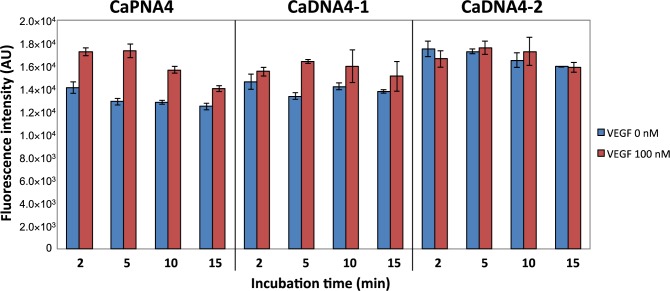
Comparison of CaPNA and CaDNAs. The fluorescein-labeled VEGF aptamer was incubated with 100 nM VEGF. The mixture was incubated with CaPNA- or CaDNA-immobilized beads. After centrifugation, the fluorescence intensity of each supernatant was measured. The blue bar shows the fluorescence intensity in the absence of VEGF. The red bar shows the fluorescence intensity in the presence of VEGF.

To evaluate the assay time required to detect VEGF in this system, we compared the capture efficiency of CaPNA4, CaDNA4-1, and CaDNA4-2 over a short time period. CaDNA4-1 is a 12-mer DNA with a *T*_m_ value similar to that of CaPNA4, and CaDNA4-2 is a 9-mer DNA (Table 1) that has the same sequence as CaPNA4. After a 2-min incubation of CaPNA or CaDNA-immobilized beads and the mixture of VEGF and the aptamer, a difference in the fluorescence intensities between in the absence and presence of VEGF was observed only for CaPNA4 (Figure 4). Furthermore, the capture efficiency of CaPNA4 was the highest of the three materials after a 5-min incubation. CaDNA4-2 failed to capture the free aptamer even after a 15-min incubation. This result indicates that the *T*_m_ value of CaDNA4-2, which was calculated to be 36°C, was too low to hybridize with the aptamer because the calculated *T*_m_ value of CaDNA4-2 is lower than that of the aptamer by about 20°C. On the other hand, CaDNA4-1 detected VEGF with a 5-min incubation. Because CaDNA4-1 has the same *T*_m_ value as that of CaPNA4, we expected CaDNA4-1 to show the same behavior as CaPNA4. However, CaDNA4-1 failed to capture the free aptamer after a 2-min incubation. Taken together, CaPNA resulted in more rapid detection of VEGF compared to CaDNA in this system.

VEGF detection systems have been commercialized and these kits can detect lower amount of VEGF than our assay. In addition, VEGF detection sensing systems using aptamers have been also reported [3,16–21]. Although an aptamer based B/F separation system using CaPNA is simple detection system, detection limit was not sufficient for the clinical applications. To improve sensitivity of this system, we need to decrease background signals, which would cause high noise for measurement. To decrease background signal, hybridization efficiency between the aptamer and CaPNA should be enhanced. Longer CaPNA might enhance hybridization between them. In this study, we postulated that VEGF binding to the aptamer would inhibit CaPNA hybridization due to steric hindrance and stabilization of the structure of the aptamer. Target molecule binding to the aptamer would stabilize not only the binding region but also whole structure, resulting in inhibition of hybridization between CaPNA. CaPNA targeting not target-binding region might increase the difference of stability between with or without target molecules.

In addition, salt concentration would affect hybridization efficiency because the structure of the aptamer would be destabilized in low concentration of salt due to negative charge repulsion between the phosphate backbones. In the lower salt concentration, hybridization efficiency between CaDNA and DNA aptamer is exactly decreased. On the other hand, the hybridization between PNA and DNA is not sensitive to salt concentration. Therefore salt concentration optimization would be effective to optimize the sensitivity of this system.

## 3. Experimental Section

### 3.1. Materials

Bovine serum albumin (BSA) was purchased from Sigma-Aldrich (St. Louis, MO, USA). VEGF was purchased from R&D Systems (Minneapolis, MN, USA). Immobilized NeutrAvidin was purchased from Thermo Scientific (Waltham, MA, USA). Biotin was purchased from Sigma-Aldrich. All oligonucleotides (Table 1) were obtained from Life Technologies (Carlsbad, CA, USA). PNAs were purchased from Panagene (Daejeon, Korea).

### 3.2. Oligonucleotides and PNAs

We used a 58-mer anti-VEGF DNA aptamer (5′-CTGGCCAGGTACCAAAAGATGATCTTGG GCCCGTCCGAATGGTGGGTGTTCTGGCCAG-3′), which contained a stem-loop structure as the VEGF recognition element, and CaPNAs (8–11-mers) to trap the VEGF aptamer. For fluorescence detection, the VEGF aptamer was labeled with fluorescein at the 5′-end. To immobilize the CaPNAs onto NeutrAvidin beads, the CaPNAs were labeled with biotin at the N-terminus.

### 3.3. Design of CaPNA

Since stem-loop2 region of the VEGF aptamer is important for VEGF recognition, each CaPNA is designed to be complementary with partial sequence of stem-loop2 region. Five CaPNAs were designed to bear the same extent *T*_m_ value of the VEGF aptamer (47°C) based on predicted *T*_m_ values calculated by the PNA probe designer [14].

### 3.4. VEGF Detection by an Aptamer Based B/F Separation System Using CaPNA

We prepared CaPNA-immobilized beads by the same way as CaDNA [3,4]. We used NeutrAvidin beads with a near-neutral isoelectric point to reduce nonspecific binding of VEGF to the beads. NeutrAvidin beads (10 μL) were washed with TBS buffer [10 mM Tris-HCl (pH 7.0), 100 mM NaCl]; the biotinylated CaPNA (160 pmol) was added to the 40-μL solution of NeutrAvidin beads and incubated for 1 h. The beads were then collected by centrifugation (5000 g, 20 s). To reduce nonspecific binding of VEGF to the beads, the beads were blocked with biotin and skimmed milk. The beads were incubated with reaction buffer that contained 4% skimmed milk and 4 mM biotin for 1 h and washed with TBS buffer. All steps were carried out at room temperature.

The fluorescein-labeled VEGF aptamer and various concentrations of VEGF or BSA were mixed with 150 μL of TBS buffer and incubated for 10 min at room temperature. The mixture was added to 10 μL of the CaPNA-immobilized beads and incubated for 5 min at room temperature. After centrifugation (5000 g, 20 s), the fluorescence intensities of the 100-μL supernatant were measured (λ_ex_ 485 nm, λ_em_ 535 nm).

## 4. Conclusions

We have successfully developed a simple and rapid B/F separation system for VEGF detection using a PNA. This simple B/F separation system is applicable for various protein-sensing systems, and can be easily constructed when a specific aptamer to the target protein is available.

Moreover, since the DNA aptamer is easily modified with an enzyme to amplify the signal, the sensitivity of this sensor system could be improved. Therefore, we consider this sensing system to have great potential for the development of easy and rapid target-molecule detection systems.

## Figures and Tables

**Table 1.** Sequences of designed CaDNAs and CaPNAs.

Name	Sequence *	*T*_m_ (°C)	Length (mer)
CaPNA1	ACCATTCGGAC	62 **	11
CaPNA2	CCCACCATTCG	59 **	11
CaPNA3	ATTCGGACGG	65 **	10
CaPNA4	ATTCGGACG	57 **	9
CaPNA5	ATTCGGAC	47 **	8
CaDNA4-1	ATTCGGACGGGC	55 ***	12
CaDNA4-2	ATTCGGACG	36 ***	9

* Directions of CaPNAs are N to C terminal; Directions of CaDNA are 5′ to 3′ terminal; ** *T*_m_ values of CaPNAs were calculated by PNA probe designer [14];

*** *T*_m_ values of CaDNAs were calculated by Oligoanalyzer [15].
